# Capecitabine plus docetaxel every 3 weeks in first- and second-line metastatic oesophageal cancer: final results of a phase II trial

**DOI:** 10.1038/sj.bjc.6602645

**Published:** 2005-06-07

**Authors:** S Lorenzen, J Duyster, C Lersch, S von Delius, M Hennig, R Bredenkamp, C Peschel, F Lordick

**Affiliations:** 1 3rd Department of Internal Medicine (Haematology/Medical Oncology), Klinikum rechts der Isar, Technical University of Munich, Ismaninger Strasse 22, Munich D-81675, Germany; 2 2nd Department of Internal Medicine (Gastroenterology), Klinikum rechts der Isar, Technical University of Munich, Ismaninger Strasse 22, Munich D-81675, Germany; 3 Institute for Medical Statistics and Epidemiology, Technical University of Munich, Ismaninger Strasse 22, Munich D-81675, Germany; 4 Munich Center for Clinical Studies, Klinikum rechts der Isar, Ismaninger Strasse 22, Munich D-81675, Germany

**Keywords:** capecitabine, docetaxel, metastatic oesophageal cancer

## Abstract

Capecitabine and docetaxel have single-agent activity in upper gastrointestinal tumours, and have together demonstrated preclinical synergy and a survival benefit in breast cancer, and high response rates in first-line metastatic gastric cancer. This trial assessed the efficacy, safety and feasibility of capecitabine in combination with docetaxel in patients with metastatic oesophageal cancer. In all, 24 patients with advanced disease (17 squamous cell carcinoma and seven adenocarcinoma) received oral capecitabine (1000 mg m^−2^ twice daily on days 1–14) plus intravenous docetaxel (75 mg m^−2^ on day 1) every 3 weeks as first- (*n*=16) or second-line (*n*=8) therapy. Patients received a median of four cycles of treatment (range, 0–6). The median follow-up is 16.5 months (range, 7.9–21.4 months). Intent-to-treat efficacy analysis showed an overall response rate of 46%. Of the 11 responders (one complete and 10 partial), nine of 16 (56%) received first-line and two of eight (25%) received second-line therapy. The median time to progression was 6.1 months (95% confidence interval (CI), 4.5–7.7 months). The meian survival was 15.8 months (95% CI, 7.8–23.9 months). Severe adverse events (grade 3/4) reported were: neutropenia (42%, including febrile neutropenia 8%), hand-foot syndrome (29%), diarrhoea (13%), sensory neuropathy (13%), anaemia (8%) and fatigue (8%). Capecitabine plus docetaxel has a manageable adverse event profile and very promising activity in metastatic oesophageal cancer, at least comparable to other doublet regimens. Therefore, the combination merits further investigation in this setting.

The incidence of oesophageal cancer is increasing (Blot *et al*, 1991) and because of early lymphogenic and haematogeneous spread, most patients are diagnosed with advanced disease. Unfortunately, patients with recurrent or metastatic disease are incurable, and palliative therapy with chemotherapeutic agents is the primary treatment option. During the last decade, numerous chemotherapeutic agents, such as cisplatin, 5-fluorouracil (5-FU), mitomycin C, irinotecan, vinorelbine and paclitaxel, have been evaluated in patients with oesophageal cancer. However, complete responses are rare, responses are usually of a short duration with a median survival of only 6–10 months (Iizuka *et al*, 1992; Bleiberg *et al*, 1997; Enzinger *et al*, 1999; Hayashi *et al*, 2001) and adverse effects, especially with cisplatin-based regimens, are often substantial. Thus, there is an urgent need for tolerable and active agents and protocols for the treatment of oesophageal cancer.

Single-agent docetaxel has revealed only moderate activity in patients with previously untreated adenocarcinoma of the upper gastrointestinal tract (Einzig *et al*, 1996; Heath *et al*, 2002), but combination regimens, for example, docetaxel plus irinotecan, have been shown to induce remissions in oesophageal cancer (Ilson *et al*, 1998, 1999; Lordick *et al*, 2003). Capecitabine is an oral fluoropyrimidine designed to generate 5-FU preferentially in tumour tissue while minimising systemic 5-FU exposure. After absorption, capecitabine is metabolised to 5-FU by a three-step process, the final step of which is mediated by thymidine phosphorylase (TP). Tumour selectivity results from the significantly greater activity of TP in tumours compared with healthy tissue (Miwa *et al*, 1998; Schüller *et al*, 2000). Taxanes result in the upregulation of TP in tumour tissue (Sawada *et al*, 1998), which appears to further enhance tumour sensitivity to capecitabine. Indeed, coadministration of capecitabine and taxanes exhibits synergistic antitumour activity in preclinical studies (Sawada *et al*, 1998; Ishitsuka, 2000). Clinically, oral capecitabine is approved and is used widely in the management of metastatic breast and colorectal cancers (Blum *et al*, 1999; Van Cutsem *et al*, 2000). As first-line therapy for metastatic colorectal cancer, capecitabine results in superior response rates, improved tolerability and greater convenience compared with 5-FU/LV (Mayo Clinic regimen) (Hoff *et al*, 2001; Van Cutsem *et al*, 2001, 2004). In pretreated metastatic breast cancer, adding capecitabine to docetaxel provides clear benefits in terms of response rates, time to disease progression and overall survival over single-agent docetaxel (O'Shaughnessy *et al*, 2002). The combination of capecitabine and taxanes has also been shown to be active as first-line treatment in phase II studies in patients with unresectable or metastatic gastric cancer (Kang *et al*, 2004; Kim *et al*, 2004). The safety profiles of capecitabine and taxanes do not appear to overlap and the combination of oral capecitabine plus docetaxel every 3 weeks can be conveniently administered as home-based therapy. Hence, we performed a phase II trial to investigate the efficacy, safety and feasibility of the combination of capecitabine and docetaxel in patients with incurable adenocarcinoma or squamous cell carcinoma of the oesophagus. As this combination was associated with a high rate of neutropenic fever in a previous study (O'Shaughnessy *et al*, 2002), we evaluated a 3-weekly regimen of capecitabine plus docetaxel at doses of 1000 mg m^−2^ twice daily on days 1–14 and 75 mg m^−2^ intravenously (i.v.) on day 1, respectively.

## MATERIALS AND METHODS

### Patient population

Patients with histologically confirmed adenocarcinoma or squamous cell carcinoma of the oesophagus or gastro-oesophageal junction were enrolled at a single site (Klinikum rechts der Isar, Technical University of Munich, Germany). The patients had to fulfil specific inclusion criteria in order to be entered on the study. To be eligible, patients had to have metastatic disease with at least one bidimensionally measurable lesion ⩾1 cm by CT scan or MRI, taken after oral and i.v. contrast, and be either chemotherapy-naive or previously treated with no more than one chemotherapy regimen. Patients had to be >18 years of age with an Eastern Cooperative Oncology Group (ECOG) performance status ⩽2 and a life expectancy of ⩾12 weeks. Adequate bone marrow, renal and hepatic function was necessary and was defined as an absolute neutrophil count ⩾1.5 × l0^9^ 1^−1^, platelets ⩾100 × 10^9^ l^−1^, serum creatinine <1.5 × upper limit of normal (ULN), total bilirubin ⩽1.5 × ULN, SGOT and/or SGPT ⩽1.5 × ULN (⩽5.0 × ULN in the presence of liver metastases). Patients were excluded if they had CNS metastases or neuropathy ⩾grade 2, as were women who were pregnant or lactating.

Complete history and physical examination, laboratory tests, CT scans of all tumour-involved areas and ECG were performed before study entry. Pregnancy testing was performed in women of childbearing age. All patients gave written, informed consent before enrolment and the study was approved by the ethics committee for human research at the Technical University of Munich. The study conformed to the principles of the Declaration of Helsinki and its subsequent amendments.

### Treatment plan

Oral capecitabine was administered at a dose of 1000 mg m^−2^ twice daily on days 1–14 every 3 weeks. Docetaxel was administered at a dose of 75 mg m^−2^ i.v. on day 1 every 3 weeks. Docetaxel was given 1 h before the first oral dose of capecitabine. Docetaxel was administered over 60 min in 500 ml saline solution. Oral dexamethasone (8 mg) was given as antiemetic prophylaxis 30 min prior to the infusion of docetaxel.

Patients underwent follow-up CT scans for assessment of response after every two cycles of therapy. Tumour response was classified on the basis of the response evaluation criteria defined by RECIST guidelines (Therasse *et al*, 2000). Responses were reviewed by independent radiologists at the Department of Radiology of the Klinikum Rechts der Isar, and were confirmed after at least 4 weeks. Patients were treated until best response or until there was evidence of disease progression, unacceptable adverse events, patient withdrawal or death. Patients were seen every week in the outpatient clinic for laboratory tests and safety evaluation, which was determined using the National Cancer Institute Common Toxicity Criteria (NCI-CTC, version 3.0).

### Statistical considerations

The primary end point of the study was to determine the proportion of patients responding to capecitabine plus docetaxel. The study was designed as a two-stage trial according to Gehan (1961), assuming a response rate of 30%. With a power of 90%, this resulted in a sample size of seven patients for the first stage. The size of the second stage was determined by the observed number of responses and by the prespecified precision of 10%. There were three responders during the first stage of the study. Therefore, according to the study design, the sample size for the whole study was extended to 24 patients. All eligible patients were included in the response, safety and survival analyses. The survival analysis was performed using SPSS software (version 12.0). Confidence intervals (CIs) (95%) were calculated for all relevant estimates using StatXact (version 5).

## RESULTS

### Patient baseline characteristic

A total of 24 patients were enrolled between February 2003 and April 2004. Patient baseline characteristics are listed in Table 1. Six (25%) of the patients had undergone oesophagectomy and half of the patients received prior radiation therapy. Of the 24 patients, 16 (67%) were chemotherapy-naive and the remaining eight (33%) had received prior chemotherapy for metastatic disease. Prior chemotherapeutic regimens mostly comprised 5-FU and platinum compound combinations. Although patients with reduced performance status (ECOG score 2) were eligible to enter the study according to the inclusion criteria, all of the treated patients were in good physical condition (ECOG scores 0 and 1).

Most of the patients had squamous cell carcinoma (71%), with lymph nodes (79%) as the main site of metastases. Other predominant sites of metastases were the lung (37%) and the liver (29%).

### Efficacy analysis

Patients completed a median of four cycles (range 0–6) of treatment. Response analysis included all of the 24 patients enrolled (intent-to-treat (ITT) population). Tumour response was evaluable according to RECIST criteria in 22 patients. Two other patients did not complete two cycles of chemotherapy, as they died before the first tumour assessment was carried out. An objective overall response was seen in 11 patients, that is, the objective response rate (ORR) was 46% (95% CI, 26–67%). There was one complete remission (CR) and 10 patients achieved partial remission (PR). Four patients had stable disease (SD) and seven patients had disease progression, primarily during the first two cycles of treatment (Table 2). The response rates in patients with no previous chemotherapy (first-line) and with previous chemotherapy (second-line) were 56% (nine of 16 (95% CI, 28–84%)) and 25% (two of eight (95% CI, 15–35%)), respectively.

The tumour control rate (ORR+SD) appeared to be associated with the two histological subtypes of oesophageal cancer: 69% (11 of 16) in patients with squamous cell carcinomas and 50% (three of six) in patients with adenocarcinomas. Notably, we discovered that three out of the eight patients treated second-line had adenocarcinomas of the oesophagus, and these patients were eventually determined to be nonresponders.

**Figure 1:**
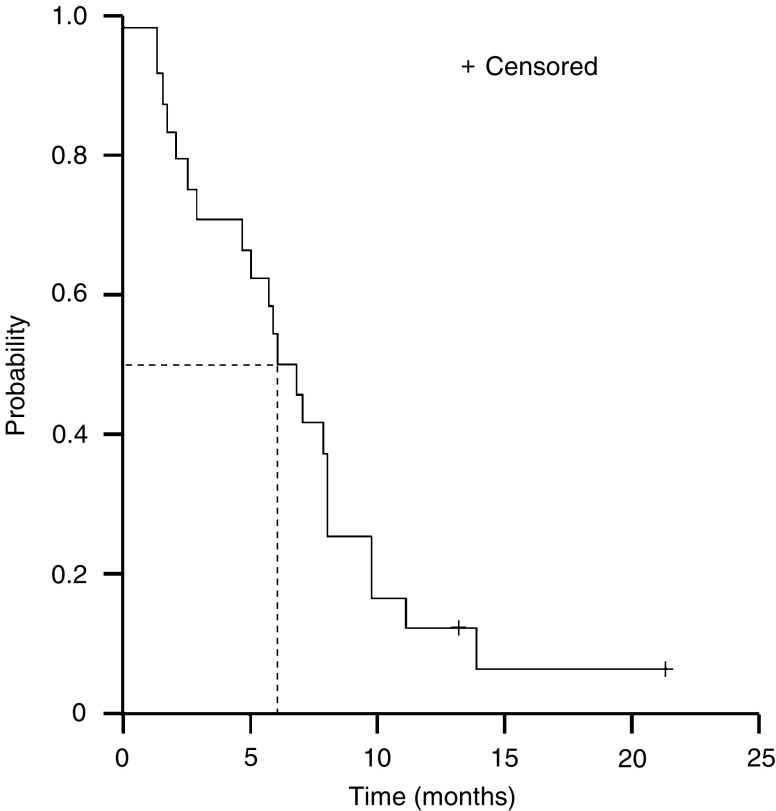
Kaplan–Meier plot of time to disease progression for all patients treated for metastatic oesophageal cancer.

**Figure 2:**
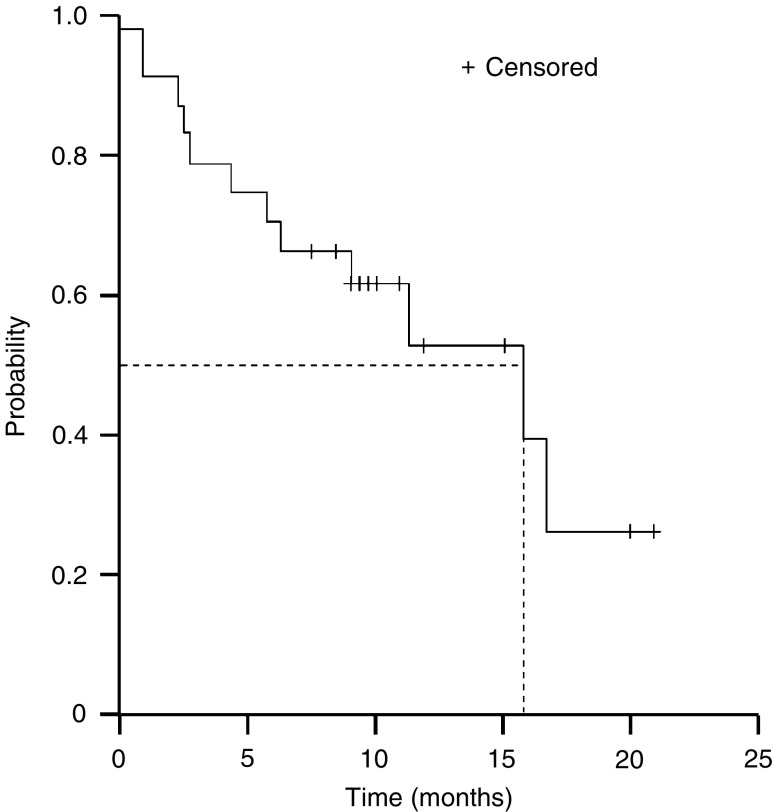
Kaplan–Meier plot of overall survival for all patients treated for metastatic oesophageal cancer.

**Figure 3:**
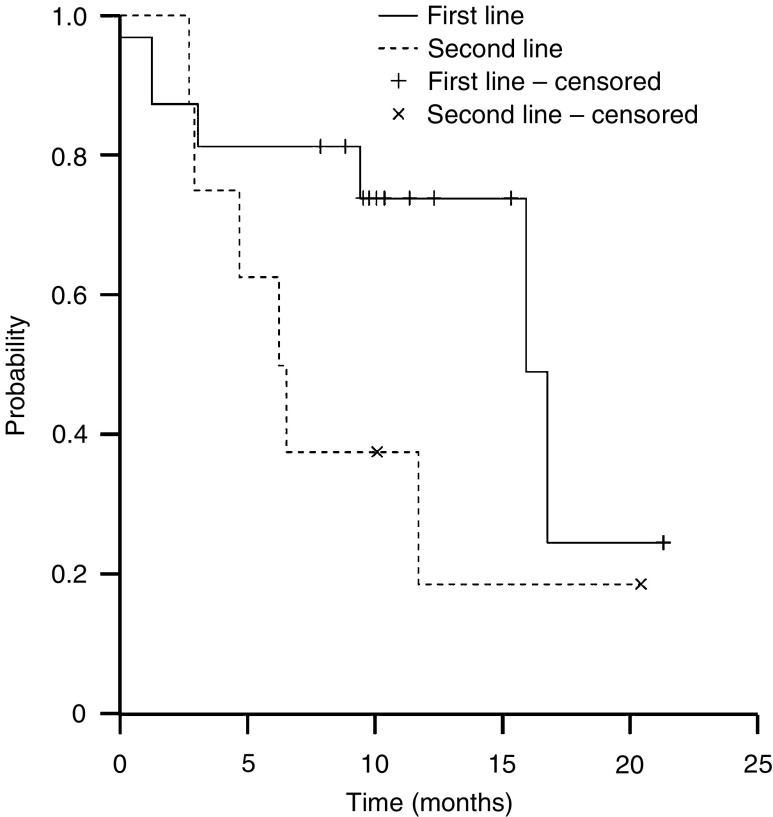
Kaplan–Meier plot of overall survival according to line of treatment for metastatic oesophageal cancer.

The median time to disease progression was 6.1 months (95% CI, 4.5–7.7 months, Figure 1). Overall survival of the 24 evaluable patients is shown in Figure 2. With a median follow-up duration of 13.4 months, the median survival time was 15.8 months (95% CI, 7.8–23.9 months). To date, 12 of the 24 patients (50%) are alive, with a median follow-up of 16.5 months (range, 7.9–21.4 months). As would be expected, median survival was longer in those who received docetaxel/capecitabine as first-line therapy (15.8 months (95% CI, 9.5–22.1 months)) compared with those treated in the second-line setting (6.2 months (95% CI, 3.5–8.8 months), Figure 3).

### Safety

Major and minor treatment-related adverse events are summarised in Table 3. Treatment was generally well tolerated in the majority of patients, with few severe adverse events. The mortality rate within the first 60 days of therapy was 8%. One patient died because of rapid tumour progression with gastrointestinal bleeding and one because of oesophageal stent perforation with subsequent mediastinitis. These early deaths were not considered to be treatment induced.

Grade 3/4 adverse events included neutropenia (42%), hand–foot syndrome (29%), diarrhoea (13%), sensory neuropathy (13%), fatigue (8%) and anaemia (8%). No episodes of grade 3/4 nausea, vomiting, stomatitis, and thrombocytopenia were reported during the study. The most common haematological toxicity of docetaxel on this schedule was neutropenia. Of the 10 (42%) patients with grade 3/4 neutropenia, two (8%) experienced febrile neutropenia with subsequent in-patient hospitalisation. The most severe nonhaematological adverse event was grade 3 hand–foot syndrome in seven patients (29%), resulting in dose reductions of capecitabine in four patients (17%) as symptoms worsened over time. Owing to adverse events, dose adjustments for one or both agents (to <80% of initial doses) were reported in 41% of patients.

## DISCUSSION

The present study was undertaken to assess the efficacy and safety of combining docetaxel with capecitabine in patients with incurable adenocarcinoma or squamous cell carcinoma of the oesophagus. With an ORR of 46% and an encouraging median survival of 15.8 months, this regimen compares favourably to other previously investigated combinations. The response rate of 56% observed in chemotherapy-naive patients is comparable to responses obtained with the commonly used combination of cisplatin plus 5-FU or the weekly treatment regimen of cisplatin and irinotecan (Ilson *et al*, 1999). As the toxicity induced by cisplatin-based chemotherapy, particularly in the palliation of metastatic disease, is often substantial, the combination of capecitabine and docetaxel seems to be an effective and feasible alternative to the standard cisplatin-based regimens for metastatic oesophageal cancer, but with less toxicity/side effects.

The results of the current study are also supported by the preliminary data of a phase II Korean study evaluating a similar regimen of capecitabine (1000 mg m^−2^ twice daily on days 1–14) and docetaxel (36 mg m^−2^ on days 1 and 8) every 3 weeks in patients with untreated metastatic gastric cancer (Kim *et al*, 2004). In that study, the ORR was 40% and median survival was 12.0 months, which is similar to that observed in the current study. In contrast to our study, where most of the patients had squamous cell carcinoma, most of the patients in the Korean study had adenocarcinoma. Nevertheless, all patients included in our study with adenocarcinoma and who were previously untreated (four of 24) were classified as responders. Another phase II study, which evaluated the activity and feasibility of first-line capecitabine (825 mg m^−2^ twice daily on days 1–14) plus paclitaxel (175 mg m^−2^ on day 1) every 3 weeks in advanced gastric cancer, showed similar results to ours, with an ORR of 53% and a median survival of 14.6 months (Kang *et al*, 2004). In contrast to the above-mentioned trials, which found the taxane–capecitabine combination to be active in oesophagogastric adenocarcinoma, the current data show for the first time the clinical efficacy of docetaxel/capecitabine in squamous cell carcinomas of the oesophagus.

While there are some promising treatment options with encouraging response and survival data in first-line metastatic oesophageal cancer, there is still a lack of effective treatment options for patients who have relapsed after a cisplatin-containing regimen. In patients with pretreated oesophageal cancer, the reported response rates vary between 0 and 16% (Conroy *et al*, 1996; Heath *et al*, 2002; Lordick *et al*, 2003; Muro *et al*, 2004). In our study, the response rate of 25% in patients with prior chemotherapy was encouraging compared to findings reported from other second-line trials. Single-agent docetaxel in pretreated patients revealed only a moderate response rate of 16% (Muro *et al*, 2004). Another trial reported that therapy with docetaxel was completely ineffective in oesophageal adenocarcinoma (Heath *et al*, 2002). However, the ability to interpret the findings from these single-agent docetaxel studies is limited by their small sample size (*n*<25). It is also important to acknowledge this same limitation in our study, with only eight patients receiving the docetaxel/capecitabine combination regimen as second-line therapy. While there is presently no standard second-line treatment for metastatic oesophageal cancer, the combination of capecitabine plus docetaxel might be an option for patients who are very keen to receive treatment.

The relative absence of grade 3/4 adverse events other than neutropenia and hand–foot syndrome seen in this trial was remarkable. Furthermore, there were no treatment-related deaths. Grade 3/4 neutropenia was the most common and severe adverse event and was observed in almost half of the patients, but only two patients (8%) developed febrile neutropenia. Similar haematological toxicities were described in other phase II studies with the combination of capecitabine and a taxane (Kang *et al*, 2004; Kim *et al*, 2004). In contrast, capecitabine plus docetaxel combination resulted in a 16% rate of neutropenic fever in second-line metastatic breast cancer (O'Shaughnessy *et al*, 2002). Consequently, in our study the dose of capecitabine was *a priori* reduced to 80% of the dose used in the metastatic breast cancer trial. Nonhaematological adverse events occurred in relatively few patients and were generally acceptable. The most frequently reported event was grade 3/4 hand–foot syndrome, affecting every third patient, which was a similar rate reported by O'Shaughnessy *et al*. This side effect was manageable with transient capecitabine interruptions or dose modifications in the majority of patients.

Gastrointestinal side effects, such as diarrhoea, nausea and vomiting, which were also anticipated with the combination, occurred less frequently than usually seen with standard dose capecitabine single-agent therapy (Scheithauer *et al*, 2003). These side effects were readily managed with appropriate medical interventions, for example, loperamide and rehydration for diarrhoea and 5-HT_3_ receptor antagonists and dexamethasone for nausea and emesis. Consistent with the known toxicities of docetaxel, neurological adverse effects occurred in a relatively high proportion of patients, although sensory neuropathy was predominantly mild to moderate in intensity. Severe grade 3/4 neurotoxicity was only reported in three patients. Generally, the combination regimen of capecitabine and docetaxel has a manageable safety profile, consistent with the known adverse events associated with the individual agents. Nevertheless, myelosuppression demands careful patient management.

In conclusion, capecitabine and docetaxel is an effective and feasible combination in the first-line treatment of metastatic oesophageal cancer and is also a promising regimen in pretreated patients. Unfortunately, the small sample size of this trial does not allow any definitive conclusions to be made about the efficacy of this combination regimen. Thus, further investigation in larger groups of patients is warranted to compare capecitabine plus docetaxel with other standard first-line chemotherapy regimens in a randomised trial and to confirm the efficacy of second-line treatment in adenocarcinoma and squamous cell carcinomas of the oesophagus.

## Figures and Tables

**Table 1:** Patient baseline characteristics

**Characteristic**	**No.**	**%**
*Age (years)*		
Median	61.5	
Range	49–72	

*Gender*		
Male	21	88
Female	3	12

*ECOG performance status*		
0	8	33
1	16	67

*Histology*		
Adenocarcinoma	7	29
Squamous cell carcinoma	17	71

*Previous treatment*		
Oesophagectomy	6	25
First-line chemotherapy	8	33
Radiotherapy	3	12
Chemoradiotherapy	9	37

ECOG=Eastern Cooperative Oncology Group.

**Table 2:** Antitumour efficacy (ITT analysis, *n*=24)

**Type of response**	**No.**	**%**	**95% CI**
ORR	11	46	26–67
CR	1	4	0.1–21
PR	10	42	22–63
SD	4	17	5–37
Progressive disease	7	29	13–51
Not evaluable	2	8	1–27

ITT=intent-to-treat; CI=confidence interval; ORR=objective response rate; CR=complete response; PR=partial response; SD=stable disease.

**Table 3:** Most common treatment-related adverse events

	**Grade 1/2**		**Grade 3/4**	
**Toxicity**	**No.**	**%**	**No.**	**%**
Anaemia	9	37	2	8
Neutropenia	3	12	10	42
Febrile neutropenia	0	0	2	8
Diarrhoea	3	12	3	13
Sensory neuropathy	7	29	3	13
Fatigue	9	37	2	8
Hand–foot syndrome	7	29	7	29
